# Toward Accurate Visual Reasoning With Dual-Path Neural Module Networks

**DOI:** 10.3389/frobt.2020.00109

**Published:** 2020-08-21

**Authors:** Ke Su, Hang Su, Jianguo Li, Jun Zhu

**Affiliations:** ^1^THBI Lab, Department of Computer Science and Technology, BNRist Center, Institute for AI, Tsinghua University, Beijing, China; ^2^Intel Labs China, Beijing, China

**Keywords:** machine learning, visual reasoning, visual question answering, neural module networks, complementary pairs

## Abstract

Visual reasoning is a critical stage in visual question answering (Antol et al., 2015), but most of the state-of-the-art methods categorized the VQA tasks as a classification problem without taking the reasoning process into account. Various approaches are proposed to solve this multi-modal task that requires both abilities of comprehension and reasoning. The recently proposed neural module network (Andreas et al., 2016b), which assembles the model with a few primitive modules, is capable of performing a spatial or arithmetical reasoning over the input image to answer the questions. Nevertheless, its performance is not satisfying especially in the real-world datasets (e.g., VQA 1.0& 2.0) due to its limited primitive modules and suboptimal layout. To address these issues, we propose a novel method of Dual-Path Neural Module Network which can implement complex visual reasoning by forming a more flexible layout regularized by the pairwise loss. Specifically, we first use the region proposal network to generate both visual and spatial information, which helps it perform spatial reasoning. Then, we advocate to process a pair of different images along with the same question simultaneously, named as a “complementary pair,” which encourages the model to learn a more reasonable layout by suppressing the overfitting to the language priors. The model can jointly learn the parameters in the primitive module and the layout generation policy, which is further boosted by introducing a novel pairwise reward. Extensive experiments show that our approach significantly improves the performance of neural module networks especially on the real-world datasets.

## 1. Introduction

**Figure 1:**
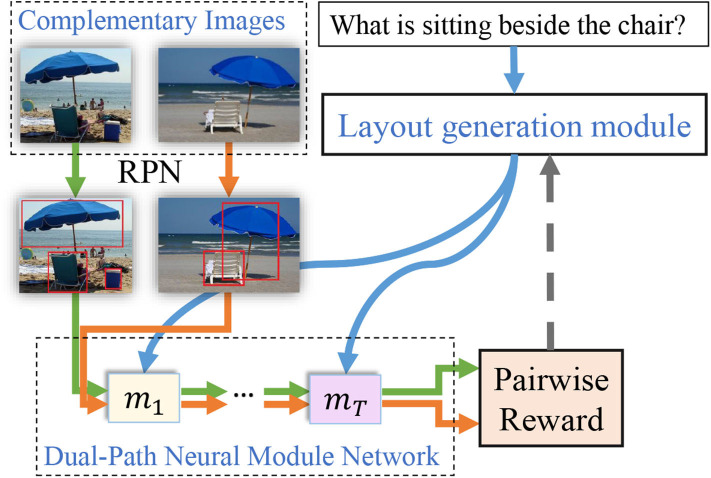
A brief illustration of our Dual-Path Neural Module Network. We input a pair of complementary images to the network along with the same question simultaneously. We propose to generate more flexible layout by regularizing the loss function with the pairwise reward. Our approach can therefore conduct more complex visual reasoning by composing the linguistic structure along with the visual and spatial information provided by the attached region proposal network (RPN).

Visual Reasoning tasks require both abilities of scene understanding and semantic reasoning of AI models to perform well. Among various visual reasoning tasks, visual question answering (VQA) is such an excellent testbed to evaluate the reasoning capability of an AI model so that it attracts more and more attention from the whole AI community for its complexity and practicability. The VQA task targets to answer language questions based on given images, so that it binds both natural language processing and visual scene understanding. Consequently, cross-modal learning ability is of vital importance for AI models to perform well on VQA tasks, where precise answers cannot be produced without a combined comprehension of both visual and semantic inputs. Some challenging questions even require human-level reasoning intelligence for answer prediction. For instance, in order to correctly answer the question “What is sitting beside the chair?” as in Figure 1, a model has to not only detect chair in the input image, but also seek for objects that lie beside the detected chair. Such capability to fully comprehend the image and question and subsequently perform complicated reasoning process is key to answer questions precisely.

Currently, VQA models apply deep neural networks to generate a combined feature representation of both visual and textual inputs. The VQA task is thereafter formalized as a classification problem where an optimized classifier is able to select for each combined feature representation a best answer from a set of candidates. Those data-driven models have achieved reasonable performances on various VQA datasets. However, as is known, VQA datasets are biased for the unavoidable correlation between questions and answers (Goyal et al., 2017), so that data-driven methods may easily overfit to language priors, and encourages VQA models to ignore input images and predict answers merely based on input questions. This flaw severely damages the learning quality of multi-modal joint embedding, which is supposed to be essential for VQA models. The problem of data bias is widely discovered and studied, as in Hudson and Manning (2019), Agrawal et al. (2016), Kafle and Kanan (2017), and Agrawal et al. (2018). To address this problem, the VQA v2.0 dataset is designed to contain complementary pairs that contain a same question and two similar images that have different answers to the assigned question. As a consequence, any VQA model that digs deep into the language bias will naturally fail on at least 50% samples in VQA v2.0 as it can't distinguish the two paired samples from each other.

Most state-of-the-art VQA methods are formulated as answer classification problem based on the joint embedding of textual and visual features. There is little relationship modeling between the question modality and image modality, so that it looks more like a black box without interpretable process. Some recent works (Fukui et al., 2016; Lu et al., 2016; Noh et al., 2016; Xu and Saenko, 2016; Ben-Younes et al., 2017; Kazemi and Elqursh, 2017; Yu et al., 2017; Anderson et al., 2018; Kim et al., 2018; Patro and Namboodiri, 2018) introduce the attention mechanism into VQA models to attend questions to salient regions of input images, so that the joint embedding of attended regions and questions carries more accurate information for question answering. With model ensemble, attention based VQA models can achieve over 72% prediction accuracy (Jiang et al., 2018) on the test set of the VQA v2.0 dataset (Goyal et al., 2017). Performance keeps rising yet an important problem remains unsolved. The lack of reasoning capability prevents human-level understanding of the decision process, and restricts the applications which require reasoning process. Besides, attention-based methods form a joint feature representation by simply fusing visual and semantic features, without considering their different roles in this multi-modal task.

Recently, Neural Module Networks (Andreas et al., 2016b; Hu et al., 2017, 2018; Johnson et al., 2017b; Mascharka et al., 2018) address the incapability of visual reasoning for traditional VQA models, and leap ahead by automatically assembling a collection of composable neural modules into an end-to-end learnable framework. To achieve this, a *network layout* is generated for each input question that represents the inner reasoning process from input to output. Primal neural modules are subsequently composed together according to the layout, forming a neural module network that takes input images as input and produce answer predictions as output. Neural module networks treat the two fundamental modes of the VQA task differently, where visual features provide sufficient information for predicting the answer, and semantic features define the specific transformation procedure from input attributes to output answer predictions. Considering the different roles of different modes of input data is of vital importance for cross-modal learning tasks that only in this way can the human understanding of multi-modal data be added naturally into the AI model. The neural module network framework provides answer explanations for human users in the form of the network layout, and achieves reasonable performance on synthetic VQA datasets like SHAPES (Andreas et al., 2016b) and CLEVR (Johnson et al., 2017a), while suffers from unsatisfied performance on large-scale real-world datasets like the VQA v2.0 dataset.

This imbalanced performance is due to three major reasons. *First*, computer rendered images has extremely limited number of objects compared with real-world photos. Incremental difficulty in detecting objects naturally adds difficulty in answering questions. *Second*, objects in real-world datasets have more complicated relationships with each other compared with generated images, where only spatial relationships are considered. *Third*, questions in real-world datasets represent more complicated reasoning process since they are annotated by human users aiming at challenging intelligence systems, while questions generated by a rule-based system have limited variations. Consequently, it's much harder to comprehend images in real-world datasets than in computer generated datasets. Specially, when integrating with attention mechanism, neural module networks should be further improved for the ability to generate precise network layouts representing input questions to achieve better performance.

To address above flaws of neural module networks, this paper proposes Dual-Path Neural Module Network (DP-NMN), which applies a novel pairwise learning schema to boost its visual reasoning capability on real-world datasets. A brief overview of our model is given in Figure 1. The basic insight is that complementary pairs in the VQA v2.0 dataset not only balance the dataset for language priors, but also have the potential to regularize and guide the training of VQA models. To achieve this goal, we introduce two novel components into the neural module networks. *First*, we reformulate the network layout generation process as a reinforcement learning problem where a policy network builds up the layout sequence step-by-step and receives a reward after the network has been assembled according to the generated layout. We make full use of the complementary pairs annotation by giving an additional pairwise reward to the policy network if the generated layout applies well for both images. This fits human intuition that layouts generated by a robust neural module network shall represent reasoning processes determined by input questions well and work on any input image. *Second*, we apply a region proposal network (RPN) on the input image to obtain salient object regions in the image, which carries sufficient visual and spatial features of detected candidate objects. This approach makes it possible for primitive modules to take both visual and spatial features as input, which naturally helps answer space related questions. We re-implemented the structures of primitive modules to ensure that the encoded information are sufficiently adopted to help perform reasoning. With the help of the region proposal network, our DP-NMN model deals with the two subtasks of low-level feature extraction and high-level reasoning separately to seek for better performance while still being interpretable. Our main contributions are three points:

- We propose a novel Dual-Path Neural Module Network (DP-NMN) model that processes input images with a region proposal network and applies a policy network to generate reasoning layout sequences;
- We present a novel pairwise learning schema that makes full use of the complementary pairs available or easily getatable in datasets to further improve performance;
- We demonstrate significant performance improvement for visual question reasoning on VQA datasets with the proposed DP-NMN.

## 2. Related Works

### 2.1. Visual Question Answering

Visual question answering requires comprehensive understanding both input questions and images for answer prediction. Recent few years have seen many newly formed datasets including hand-crafted datasets with computer generated images, like SHAPES (Andreas et al., 2016b), CLEVR (Johnson et al., 2017a), and large-scale real-world datasets like VQA (Antol et al., 2015) and VQA v2.0 (Goyal et al., 2017). Visual reasoning is of vital importance to perform this task well, for questions are designed to contain complicated reasoning process. For example, questions in CLEVR tend to query about relationships between objects, which require VQA models to comprehend the complex relationships between detected objects. The visual attention mechanism has been widely applied to form joint representations of input questions and images, which are subsequently handled by a classifier to produce answer predictions. Recent years have seen significant improvement in terms of performance, by either enhancing the visual attention module (Xu and Saenko, 2016; Yang et al., 2016; Kazemi and Elqursh, 2017; Anderson et al., 2018; Patro and Namboodiri, 2018), or improving quality of the joint embedding (Fukui et al., 2016; Lu et al., 2016; Noh et al., 2016; Ben-Younes et al., 2017; Yu et al., 2017). With model ensemble, the current state-of-the-art model has achieved over 72% accuracy (Jiang et al., 2018) on the VQA v2.0 test set.

However, VQA models based on the visual attention mechanism are not able to provide a thorough explanation of the reasoning process from input to output. Consequently, it remains unclear whether the model truly has the ability to understand the multi-modal input to make complicated reasoning, or the model just simply overfits the dataset. The language bias problem is discovered and discussed in Goyal et al. (2017), which reveals that data-driven models may easily overfit to the unavoidable language priors between questions and answers. In contrast, the neural module network architecture takes a step ahead in visual reasoning that the generated network layout for each input question is by itself a well defined explanation of the inner reasoning process. But the performance of neural module networks on real-world VQA datasets is unsatisfied, which severely damages its practicability.

## 3. Neural Module Networks

Neural module networks (NMN) (Andreas et al., 2016b; Hu et al., 2017, 2018; Johnson et al., 2017b; Mascharka et al., 2018; Vedantam et al., 2019) provide a general idea of composing a new deep network with neural modules for each given input. Specifically for the VQA task, a structure layout for neural modules is generated based on semantic analysis on the input question, which represents the reasoning process from input image-question pair to output question answers. Subsequently, composable modules that each represents a unit step of visual reasoning are assembled together according to the generated layout, yielding a neural module network that is able to process images and predict answers. Those primitive modules have hand-crafted structures designed by human experts.

Recently, Hu et al. (2017) present the *End-to-End Module Network* which seeks for optimal layouts by predicting coarse functional expressions given input questions which describe desired network layouts. The layout generation problem is formulated as a sequence-to-sequence learning problem, and can be trained end-to-end with gradient backpropagation. This framework achieved reasonable results on simple VQA datasets like the SHAPES dataset, but suffered from relatively low accuracy on large-scale real-world VQA v2.0 dataset. Yet neural module networks still have demonstrated the advantages in interpretability of the visual reasoning process. Therefore, it remains an important direction to boost the performance for neural module networks and close the performance gap to state-of-the-art VQA methods.

## 4. Methods

In this paper, we propose Dual-Path Neural Module Network which processes pairwise data samples in parallel, and is trained with a novel pairwise learning schema. An overview of our model is provided in section 3.1. The implementation details of our model are described in section 3.2. Optimization methods applied for training our model are described in section 3.3. Our model contains several types of composable primitive modules, which are introduced in section 3.4.

### 4.1. Overview

VQA datasets contain triplet samples *s*_*i*_ = (*Q, I, A*) where *Q* denotes the input question, *I* denotes the input image and *A* denotes the ground truth answer to the question. Like previous neural module networks, our model first generates a network layout *L*_*q*_ based on the input question *Q*, and assembles a neural module network with primitive modules according to *L*_*q*_. Those primitive modules are parameterized by θ_*N*_. Then the assembled network is capable of making answer predictions given input images.

However, VQA models that works merely on singular samples tend to easily overfit to the language priors in the dataset, focusing on the strong relationship between questions and images. The VQA v2.0 dataset contains complementary pairs to address this problem, where two paired samples have a same question but different images and answers, which can be denoted as *s*_*i*_ and *s*_*j*_, where $s_{j}=\left(Q,I^{\prime },A^{\prime }\right)$ has the same question *Q* as *s*_*i*_.

In order to minimize the impact of language priors in VQA datasets and generate comprehensive network layouts, our Dual-Path Neural Module Network applies a novel pairwise learning schema that makes full use of complementary pairs. Input to our model are paired samples *s*_*i*_ and *s*_*j*_ instead of singular samples. Since the relationship between network layout and answer prediction is non-differentiable, we apply a policy network to produce layouts given input questions, which is trained under a reinforcement learning environment. During training, a reward *R*(*L*_*q*_, θ_*N*_|*s*) is given to the policy network if the predicted answers fits the ground truth answers for each sample *s*.

Our goal is to find optimal *L*_*q*_ and θ_*N*_ that not only make most accurate answer predictions, but also produce more comprehensive layouts. Intuitively, comprehensive network layouts shall work well on both complementary samples *s*_*i*_ and *s*_*j*_, making precise answer predictions for both images. Therefore, a pairwise reward Ω(*L*_*q*_, θ_*N*_|*s*_*i*_, *s*_*j*_) is defined on each pair that gives the model additional reward if the predictions are correct on both paired images to encourage robust layouts. During training, we jointly optimize the task reward for answer accuracy and the pairwise reward for regularization. Hence the optimization objective of our model can be formalized as:

(1) $$\begin{array}{l} \left(L_{q},\theta_{N}\right)=\underset{L_{q},\theta_{N}}{\text{arg max}}(\underset{s=s_{i},s_{j}}{\sum }R\left(L_{q},\theta_{N}|s\right)+\lambda \Omega \left(L_{q},\theta_{N}|s_{i},s_{j})\right), \end{array}$$

where network layout *L*_*q*_ and module parameters θ_*N*_ are jointly optimized to achieve highest reward. The network layout *L*_*q*_ is determined by two factors: types of primitive modules and connections between those modules. In practice, *L*_*q*_ is generated via the layout generator, which is optimized jointly with module parameters θ_*N*_ using this equation. Suppose that function *g* builds a network structure with a sequence *l* containing type information of all modules and a matrix Ψ that describes connections between them:

(2) $$\begin{array}{l} L_{q}=g\left(l,\Psi \right). \end{array}$$

**Figure 2:**
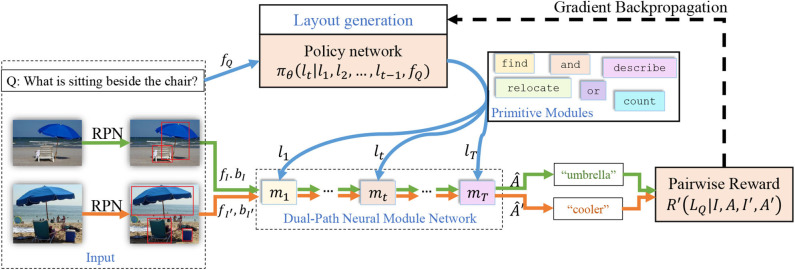
An overview of our model. The input question is fed into the layout generation module which selects one module from all possible primitive modules at each time step to assemble a neural module network, and the paired input images are fed into the dual-path neural module network which processes them in parallel. A pairwise reward is calculated for each pair and given to the layout generation module to encourage layouts that work for both images.

Hence given a sequence *l* of all type information of modules and matrix Ψ denoting the connections between them, a network layout *L*_*q*_ can be determined by function *g*. An overview of our model is shown in Figure 2. Details of the model architecture and the training method will be discussed in later sections.

### 4.2. Model Architecture

Our model composes a neural module network and predicts an answer (Â) out of a set of candidate answers for each given input pair of image (*I*) and question (*Q*):

(3) $$\begin{array}{l} Â=F\left(r_{I};L_{q},\theta_{N}\right), \end{array}$$

where *r*_*I*_ are region proposals that consist of visual features and corresponding bounding boxes of all candidate objects detected in the input image *I*. In this paper, we detect top 36 salient regions in *I* as candidate objects, each corresponds with a 2, 048 dimension visual feature vector and a 6 dimension spatial feature vector. Hence dimension for *r*_*I*_ is (36 ∗ 2048 + 6). Textual feature of input question is also extracted, denoted as *f*_*q*_ which is a 2, 048 dimension vector:

(4) $$\begin{array}{l} r_{I}=RPN\left(I\right),\text{and }f_{q}=RNN\left(Q\right), \end{array}$$

where *RPN* denotes the pre-trained region proposal network that extracts visual and spatial features of salient regions and *RNN* denotes a recurrent neural network (Bahdanau et al., 2014) that extracts textual features from the input question. The probability of any candidate answer given input question and image is divided into two parts. Firstly, a network layout *L*_*q*_ is generated based on the input question. Then composable primitive modules can be assembled together to form a neural module network, which is able to output answer predictions given input images.

Recall that in order to determine a layout *L*_*q*_, the type information of all modules *l* and the connections between those modules Ψ shall be settled. The layout sequence *l* = *l*_1_, *l*_2_, …, *l*_*T*_ is generated based on textual feature of the input question, *f*_*q*_. For each *t*, the corresponding element *l*_*t*_ in the layout sequence represents a module type among all types of primitive modules. Our model generates the layout sequence *l* step-by-step according to a policy network π_θ_, where θ denotes its parameters. At each time step *t*, the policy network produces a probability distribution π_θ_(*l*_*t*_|*l*_1_, …, *l*_*t*−1_, *f*_*q*_) for all possible types of neural modules based on textual feature of the input question and previous generated modules. The most possible module is selected to be the *t*-th neural module *l*_*t*_ to form the whole layout sequence *l*, where each *l*_*t*_ is generated by the policy network:

(5) $$\begin{array}{l} l_{t}=\underset{l_{t}}{\text{arg max}}\pi_{\theta }\left(l_{t}|l_{1},\ldots ,l_{t-1},f_{q}\right),\;t=1,2,\ldots ,T. \end{array}$$

This makes it possible to apply beam search during both training and testing to form an optimal *l* with highest probability. As in Hu et al. (2017), the layout sequence is mapped into a network layout with possible tree structure using Reverse Polish Notation, which is equivalent to Ψ. Hence after the layout sequence *l* is confirmed, we are able to apply Equation (2) to form a network layout *L*_*q*_ and subsequently assemble neural modules according to *L*_*q*_. Each neural module is a function that takes 0, 1, or 2 attention maps and optional visual and textual feature as input, and outputs either an attention map or a probability distribution for all candidate answers. It's safe to assume that the first *T* − 1 neural modules work together to output an attention map *a*, which is taken as input by the last module to form the final answer prediction. The overall function of the first *T* − 1 modules may be summed up as one function *m*_1…*T* − 1_, and the last module as *m*_*T*_. Then the answer prediction process can be formalized as:

(6) $$\begin{array}{l} a=m_{1\ldots T-1}\left(r_{I};\theta_{N}\right), \end{array}$$

(7) $$\begin{array}{l} F\left(r_{I};L_{q},\theta_{N}\right)=softmax\left(m_{T}\left(a◦r_{I};\theta_{N}\right)\right), \end{array}$$

where θ_*N*_ denotes parameters of primitive modules, and ◦ denotes element-wise multiplication between attention weights and visual features. In conclusion, upon each input pair of question and image, our model first assembles a neural module network step-by-step conditioned on the question. Subsequently, the assembled neural module network is able to take images as input and predict answer probabilities.

### 4.3. Model Optimization

The prediction accuracy of neural module networks is directly related to two separated parts of model: layout generation and neural modules. Here we apply a E-M method to train the two parts alternately. That is, we first initialize layouts with a rule-based system as in Hu et al. (2017), and train the neural modules with simple backpropagation, resulting in optimal parameters θ_*N*_. Then we fix the neural modules and train the layout generation module with policy gradient, leading to an optimal layout *L*_*q*_. This process can be repeated until we reach the joint optimum of θ_*N*_ and *L*_*q*_.

During training, we feed pairwise inputs *s*_*i*_ = (*Q, I, A*) and $s_{j}=\left(Q,I^{\prime },A^{\prime }\right)$ together into the Dual-Path Neural Module Network, which processes the two data samples in parallel. The network layout *L*_*q*_ has a non-differential relationship with the prediction accuracy, therefore the policy network π_θ_ cannot be trained directly with back propagation. However, when the whole layout is generated, which means the predicted answers Â and Â′ become accessible by feeding the input images *I* and *I*′ into the assembled network, we are able to compare the predicted answers with ground truth answers *A* and *A*′. We can define a reward function representing the result of this comparison. Optimizing this reward is thus beneficial to improving prediction accuracy. The reward function on pairwise data samples can be defined as:

(8) $$\begin{array}{l} R\left(L_{q},\theta_{N}|s_{i}\right)=1\left[Â=A\right], \end{array}$$

(9) $$\begin{array}{l} R\left(L_{q},\theta_{N}|s_{j}\right)=1\left[{Â}^{\prime }=A^{\prime }\right], \end{array}$$

where Â and Â′ are predicted answers for *I* and *I*′ correspondingly. Note that here both answer predictions Â and Â′ are generated by the neural module network that is assembled according to layout *L*_*q*_. 1[Â = *A*] is the indicator function that equals 1 if and only if the condition Â = *A* is satisfied. That is,

(10) $$\begin{array}{l} 1\left[Â=A\right]=\{\begin{array}{l} 1,\;Â=A,\\ 0,\;Â\neq A. \end{array} \end{array}$$

And same equation stands for 1[Â′ = *A*′]. Those indicator functions compare predicted answers with ground truth answers, ensuring that optimizing this task reward is beneficial to the prediction accuracy of the model. But as we have claimed, models trained with merely task reward may easily overfit to language priors in the dataset. For neural module networks, this means that layouts generated for input questions may be under-qualified to process visual reasoning on input images, but instead fit the correlations between questions and answers. To address this problem, our Dual-Path Neural Module Network applies a novel pairwise reward Ω(*L*_*q*_, θ_*N*_|*s*_*i*_, *s*_*j*_) for pairwise input samples as additional regularization during the training process. The pairwise reward is designed to encourage comprehensive layouts that work well on both paired inputs:

(11) $$\begin{array}{l} \Omega \left(L_{q},\theta_{N}|s_{i},s_{j}\right)=1\left[Â=A\wedge {Â}^{\prime }=A^{\prime }\right], \end{array}$$

where *s*_*i*_ and *s*_*j*_ are paired inputs that share a same question. It's intuitive that this pairwise reward is given to the model only when it's capable of answering the question *Q* correctly on both complementary images *I* and *I*′. Therefore, optimizing this pairwise reward efficiently avoids overfitting to language priors, for a VQA model must be able to distinguish the two complementary samples *s*_*i*_ and *s*_*j*_ to get this pairwise reward, which is nearly impossible for overfitted models that predict answers based on questions only. During training, we combine task rewards and the pairwise reward to form a total reward:

(12) $$\begin{array}{l} \begin{array}{c} R\left(L_{q},\theta_{N}\right)=\underset{s=s_{i},s_{j}}{\sum }R\left(L_{q},\theta_{N}|s\right)+\lambda \Omega \left(L_{q},\theta_{N}|s_{i},s_{j}\right)\\ =1\left[Â=A\right]+1\left[{Â}^{\prime }=A^{\prime }\right]+\lambda 1\left[Â=A\wedge {Â}^{\prime }=A^{\prime }\right], \end{array} \end{array}$$

where λ serves as a weight factor, which is set to 0.1 in this paper. Optimizing this total reward not only improves prediction accuracies on singular data samples, but also avoids overfitting to language priors. Note that at this step, θ_*N*_ is set to be fixed, so that it can be omitted from the reward function. Therefore, we optimize the total expected reward over θ to seek for optimal parameters of the policy network:

(13) $$\begin{array}{l} \theta^{*}=\underset{\theta }{\text{arg max}}E_{L_{q}}\left[R\left(L_{q}\right)\right)]. \end{array}$$

It's clear that optimal layout *L*_*q*_ is equivalent to optimal θ. However, there is no close-form solution for this optimization problem since the reward is non-differentiable with regard to the layout *L*_*q*_. To simplify the optimization, we use Monte-Carlo sampling to calculate an unbiased estimation of the expected reward:

(14) $$\begin{array}{l} \begin{array}{c} E_{L_{q}}\left[R\left(L_{q}\right)\right]\approx \frac{1}{N}\overset{N}{\underset{n=1}{\sum }}R\left(L_{q}^{\left(i\right)}\right), \end{array} \end{array}$$

where *N* denotes the number of samples drawn, and $L_{q}^{\left(i\right)}$ denotes the *i*-th sampled layout. Each sampling process requires the policy network π_θ_ to produce all module types to form a complete layout $L_{q}^{\left(i\right)}$. Then the gradients for training can be computed using policy gradient method, where we sum up the gradients at each time step:

(15) $$\begin{array}{l} \nabla E_{L_{q}}\left[R\left(L_{q}\right)\right]\approx \frac{1}{N}\overset{N}{\underset{i=1}{\sum }}\overset{T}{\underset{t=1}{\sum }}\nabla_{\theta }log\left(\pi \left(l_{t}|l_{1},\ldots ,l_{t-1},f_{q}\right)\right)R\left(L_{q}^{\left(i\right)}\right). \end{array}$$

Then we are able to train the policy network with gradient backpropagation. After the policy network is trained, which indicates that we've already reached optimal *L*_*q*_ under current module parameters θ_*N*_, we can fix π_θ_ and alternate the training process to train the parameters of composable primitive modules:

(16) $$\begin{array}{l} \theta_{N}^{*}=\underset{\theta_{N}}{\text{arg max}}R\left(L_{q},\theta_{N}\right), \end{array}$$

where *L*_*q*_ is set to be fixed during this process, hence the reward function is equivalent to a simple loss function that is differentiable with regard to θ_*N*_, and can be directly optimized with backpropagation. In this paper, we apply one iteration of this E-M process to search for the joint optimum of *L*_*q*_ and θ_*N*_.

### 4.4. Primitive Modules

As is claimed before, with the help of the region proposal network, we are able to design several new types of neural modules that take not only visual features as input, but also take spatial features into consideration. We list all types of primitive neural modules in Table 1. Note that some of those modules are declared in previous works, but are not applied for VQA v2.0.

**Table 1:** Definitions of composable neural modules.

**Module name**	**Inputs**	**Output**	**Implementation**
find	**f**_*q*_, **r**_*I*_	*att*	*a*_*o*_ = *conv*(*W*_*r*_**r**_*I*_ ⊙ *W*_*q*_**f**_*q*_)
relocate	*a*, **f**_*q*_, **r**_*I*_	*att*	*a*_*o*_ = *conv*(*W*_*v*_**v** ⊙ *W*_*q*_**f**_*q*_)
and	*a*_1_, *a*_2_	*att*	*a*_*o*_ = *min*(*a*_1_, *a*_2_)
or	*a*_1_, *a*_2_	*att*	*a*_*o*_ = *max*(*a*_1_, *a*_2_)
describe	*a*, **f**_*q*_, **r**_*I*_	*ans*	$ans=W_{a}^{T}\left(W_{v}\text{v}⊙W_{q}{\text{f}}_{q}\right)$
compare	*a*_1_, *a*_2_, **f**_*q*_, **r**_*I*_	*ans*	$ans=W_{a}^{T}\left(W_{v}\left[{\text{v}}_{1};{\text{v}}_{2}\right]⊙W_{q}{\text{f}}_{q}\right)$

*Note that with the help of the region proposal network, we are able to provide **r**_I_ as input for primitive modules which encodes both visual and spatial features. Among implementations, [p; q] denotes concatenation of two vectors p and q, and ⊙ denotes element-wise multiplication. Vectors **v**, **v**_1_, and **v**_2_ are attended visual features that are weighted sum of the region proposal **r**_I_, weighted by a_1_ and a_2_, respectively. That is, ${\text{v}}_{1}=\underset{i}{\sum }a_{1}^{\left(i\right)}{\text{r}}_{I}^{\left(i\right)}$, and $v_{2}=\underset{i}{\sum }a_{2}^{\left(i\right)}{\text{r}}_{I}^{\left(i\right)}$, where $a_{1}^{\left(i\right)}$ denotes the i-th component of the input attention map, and ${\text{r}}_{I}^{\left(i\right)}$ denotes the i-th row of the visual feature encoded in **r**_I_. Note that model parameters W_q_, W_r_, W_v_, W_a_ are not shared among different modules, which are parameters related to textual features, region proposals, visual features, and attention maps, respectively*.

It's also worth noting that since we adopt a region proposal network to extract candidate objects and corresponding bounding boxes of them, not only noisy redundant information are filtered, but also additional information is provided to the composed neural module network. Neural modules are now able to take spatial information as input, in addition to visual and textual features. For example, being able to take *r*_*I*_ as input, which encodes both visual and spatial information, directly helps the relocate module to solve space-related questions like “to the left of,” “inside,” and “bigger than,” which contributes to the performance improvement. Previous works adopt rule-based systems to parse each question sequence in the training set to automatically generate layouts for initialization during training. Since our model contains several new types of neural modules, we modify the question parser to generate more suitable layouts, especially for space related questions. As a consequence, the neural module network will generate more comprehensive layouts on those questions and achieve higher accuracy during testing.

## 5. Experiments

We evaluate the proposed method on the test sets of VQA v2.0 (Goyal et al., 2017) and CLEVR (Johnson et al., 2017a). Similarly to previous works, we pre-process the question sentences using pre-trained GloVe (Pennington et al., 2014) vectors with 300 dimensions. The GloVe representations are fed into a bi-layer LSTM with hidden dimensions set as 1,000. The input image is pre-processed with a ResNet (He et al., 2016) which outputs 2,048-dimension feature representations of input images. Our model is implemented using TensorFlow (Abadi et al., 2016), and is trained with Adam Optimizer (Kinga and Adam, 2015), with learning rate set as 1 × 10^−4^ and β set as 0.99. We compare performances of our DP-NMN with the baseline model End-to-End Neural Module Network (N2NMN) (Hu et al., 2017), and also several attention-based models. We also give qualitative analyses of generated network layouts. During training, batches of size 64 are fed into the model for 80, 000 iterations. The training process will terminate when the validation performance stays unimproved for 5, 000 iterations.

### 5.1. Datasets

#### 5.1.1. VQA v2.0

VQA v2.0 is a VQA dataset that minimizes the impact of language bias with pairwise data samples, containing over 1.1 M human annotated questions and 0.4M MSCOCO (Lin et al., 2014) images. Faster-RCNN (Ren et al., 2015) is applied as the region proposal network that detects candidate objects, which uses a ResNet (He et al., 2016) CNN trained on ImageNet (Russakovsky et al., 2015) as visual feature extractor. We directly adopt the pre-trained Faster-RCNN available in Anderson et al. (2018). As in Hu et al. (2017), the layouts are firstly initialized with pre-generated layouts in Andreas et al. (2016a). Then we use the policy gradient method described in section 3.3 to search for better layouts. Accuracies on VQA v2.0 are reported by EvalAI (VQA, 2016).

#### 5.1.2. CLEVR

The CLEVR (Johnson et al., 2017a) dataset focuses on relational reasoning, and contains 700K, 150K, and 150K automatically generated questions for training, validation and testing. We fine-tune a VGG-Net (Simonyan and Zisserman, 2014) to provide visual features. No complementary annotations are directly available to train our DP-NMN, which makes it impossible to adopt our pairwise learning schema out of the box. Fortunately, since questions in CLEVR have limited grammar structures, we may pre-process questions in the training set such that words representing a same concept are replaced with a same placeholder. For example, all words describing colors are replaced with placeholder “{color}.” Similar replacements are applied for all words describing sizes, shapes, materials, and directions.

After pre-processing, we are able to extract complementary pairs. Two identical questions after the replacement indicate that they represent a same reasoning process, although specific semantic meanings of the two questions may differ. For example, “What color is the cube to the right of the yellow sphere?” and “What color is the sphere to the left of the red cylinder?” refer to different colors, directions and shapes, but the layout structure generated by NMN shall be identical. Therefore these two questions are assigned pair with each other. Practically, we are able to extract 258,329 complementary pairs (516,658 data samples) this way, covering 74% of the training set. After that, all unpaired questions are assigned pair with themselves. We are hereafter able to train our DP-NMN with pairwise learning.

### 5.2. Layout Complexity Analysis

The ability to perform complex reasoning process of neural module networks can be measured with the complexity of the generated layouts. Table 2 shows the average size of generated layouts of our model and N2NMN (Hu et al., 2017) on the test-dev set of VQA v2.0. The size of a reasoning layout is measured by the number of primitive modules it contains. For example, size of the layout find→describe is 2 for it contains two modules. It's shown that layouts generated by our model are 12% larger in size than those generated by N2NMN. Longer layouts indicate that when solving a same question, our model is able to perform more complicated reasoning process on the input image, which naturally leads to performance improvement.

**Table 2:** Average size of generated layouts, measured by number of modules, on the test-dev set of the VQA v2.0 dataset.

**Model**	**Average size of layouts**
N2NMN (Hu et al., 2017)	2.79
DP-NMN (Ours)	**3.14**

*The bold values indicate the parameters or results that belong to our model DP-NMN*.

**Figure 3:**
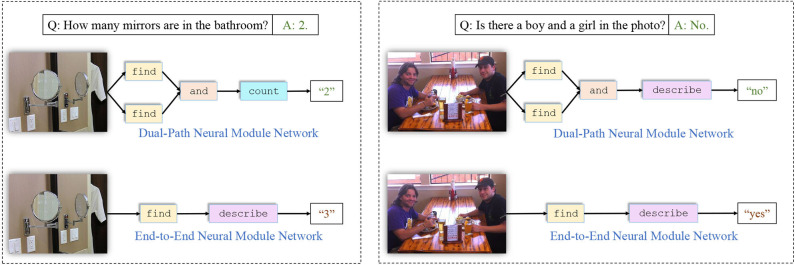
An qualitative comparison between our Dual-Path Neural Module Network and End-to-End Neural Module Network. Our policy network generates more comprehensive layouts that represent the visual reasoning process more precisely.

**Figure 4:**
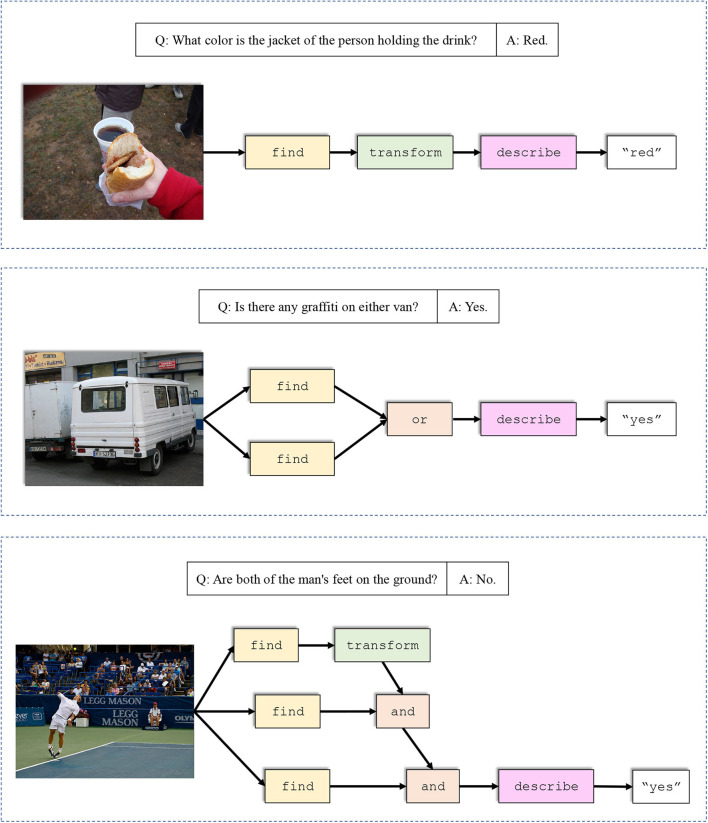
Several examples of the generated layouts by our DP-NMN. First two examples show that our DP-NMN is capable of performing spatial reasoning with the transform module, and logical reasoning with the or module. In the third example, our DP-NMN generates a complicated layout without clear meaning and outputs a wrong answer, which may indicates that the question is beyond its capability.

We also give a few examples of network layouts generated by our model together with the corresponding input questions and images to further demonstrate that our model produces more comprehensive layouts than former neural module networks like N2NMN. The results are shown in Figure 3. The presented questions and images are selected from the test set of VQA v2.0. It can be seen that our model answers more accurately, either by applying specific modules to solve particular questions, or by assembling more complex layouts to get a better understanding of the scene. In Figure 4, we give three more examples of the generated layouts by our DP-NMN. When given simple questions requiring either spatial or logical reasoning, our DP-NMN is capable of generate proper layouts of neural module networks that precisely represent the inner reasoning process. However, there are also cases observed that when the input question seems too complicated, the corresponding layout has complex structure and no clear meaning can be obtained. This may indicate that the reasoning capability of such neural module networks is still limited.

### 5.3. Benchmark Results

We report benchmark performance of our model on the test sets of VQA v2.0 and CLEVR in Table 3. Compared with the baseline model End-to-End Neural Module Network (N2NMN), it's shown that our model outperforms it by a large margin on both VQA v2.0 and CLEVR. We also compare single model performances of our model with several attention based models, including Bottom-Up and Top-Down Attention (BUTD) (Anderson et al., 2018) which took the lead in VQA Challenge 2017 (VQA, 2016). Our model outperforms BUTD on VQA v2.0, while providing better interpretability for human users. With model ensemble, our DP-NMN is able to achieve over 70% prediction accuracy. The ensemble is done by independently train 9 models at once, where they have same settings except with different random seeds. The ensemble output is given by major vote.

**Table 3:** Prediction accuracies on test sets of VQA v2.0 and CLEVR.

**Model**	**VQA v2.0**				**CLEVR**
	**Overall**	**Yes/No**	**Number**	**Others**	
Attention based models:					
MCB (Fukui et al., 2016)	62.27	78.82	38.28	53.36	51.4
BUTD (Anderson et al., 2018)	66.40	79.32	39.77	52.59	94.5
BAN-12 (Kim et al., 2018)	69.52	85.31	50.93	60.26	–
LXMERT (Tan and Bansal, 2019)	72.5	88.2	54.2	63.1	–
Neural module networks:					
N2NMN (Hu et al., 2017)	63.30	80.89	39.82	53.50	83.7
DP-NMN (Ours, Random Pairs)	66.21	83.34	43.00	56.89	90.1
DP-NMN (Ours, λ = 0)	66.37	83.43	43.81	56.95	–
DP-NMN (Ours)	**67.15**	84.37	44.83	57.50	**92.0**
DP-NMN (Ours, 9 ensemble)	**70.10**	87.24	51.53	61.09	**94.1**

*Our model significantly improves performance on VQA v2.0 compared with former neural module networks, and has comparable performance with attention based models. On CLEVR, our pairwise learning schema also shows positive impact on accuracy*.

*The bold values indicate the parameters or results that belong to our model DP-NMN*.

This reveals that the neural module network architecture is fully capable of achieving reasonable performance on large-scale real-world datasets. It can be seen that there still remains performance gap between well-designed attention-based VQA models [like BAN (Kim et al., 2018) and LXMERT (Tan and Bansal, 2019)] and neural module networks, probably showing the widely-discovered trade-off between interpretability and performance. It's also worth noting that neural module networks have the advantage over attention based models that they provide better explanations to human users when answering questions, thus being more interpretable.

We also propose experiments where randomly assigned pairs are used to train our DP-NMN, namely Random Pairs in Table 3, aiming to validate the effectiveness of the pairwise learning schema. It's clearly shown that when complementary pairs are randomly assigned, performance of DP-NMN drops by a small margin, proving that the proposed pairwise learning schema only works with well designed complementary pairs.

As shown in Table 3, the DP-NMN (λ = 0) model processes pairwise data samples but without receiving the pairwise reward. It's clearly visible that its performance drops by a large margin compared to the DP-NMN, which indicates that the pairwise reward affects the model's performance positively. Compared to the Random Pairs setting, DP-NMN (λ = 0) achieves slightly better performance. This performance gap may be related to the structure of training batches, where pairwise samples are always shown to the model simultaneously.

The selection of the parameter λ is find challenging for DP-NMN. Big values of lambda affect the training loss too much that negative influence on performance are discovered. Small values of lambda make the benefits of the proposed pairwise learning schema less viable. Therefore, we finally choose 0.1 as the most proper value of lambda. The detailed sensitivity analysis results of λ is shown in Table 4.

**Table 4:** Sensitivity analysis results of λ.

λ	1	0.5	**0.1**	0.01	0.001	0
Valid accuracy (%)	57.27	64.39	**65.21**	63.84	63.42	63.40

*Reported are validation accuracies on VQA v2.0 for different values of λ. The impact of λ can be clearly shown in the table that the trade-off between optimizing task reward and pairwise reward shall be balanced to achieve best performance on the validation split. According to the sensitivity analysis, we finally set λ as 0.1*.

*The bold values indicate the parameters or results that belong to our model DP-NMN*.

## 6. Conclusion

In this paper, we propose Dual-Path Neural Module Network that aims at better visual question reasoning on large-scale real-world datasets by introducing a novel pairwise learning schema. Our model processes the complementary images in parallel to produce a pairwise reward during the training process, which encourages to generate more comprehensive layouts of reasoning modules. Besides, we adopt a region proposal network to detect visual and spatial features of candidate objects in the input image, which provides useful spatial information for the assembled neural module network. Experimental results show that our model significantly outperforms previous neural module networks on real-world datasets, and also generalizes well on other datasets. The pairwise learning schema can be applied only when proper annotations of complementary samples are given along with the dataset, or can be easily extracted, which becomes a limitation of the framework. However, we believe that the idea of adopting those kind of complementary information to serve as additional guidance during the training process of neural networks is promising, and we expect further studies from the deep learning community.

## Data Availability Statement

All datasets generated for this study are included in the article/supplementary material.

## Author Contributions

This work is mainly done by KS under the supervision of HS, JL, and JZ. All authors contributed to the article and approved the submitted version.

## Conflict of Interest

JL was employed by the company Intel Labs China. The remaining authors declare that the research was conducted in the absence of any commercial or financial relationships that could be construed as a potential conflict of interest.

## References

[B1] Abadi M. Barham P. Chen J. Chen Z. Davis A. Dean J. (2016). Tensorflow: a system for large-scale machine learning, in OSDI, Vol. 16, 265–283.

[B2] Agrawal A. Batra D. Parikh D. (2016). Analyzing the behavior of visual question answering models. arXiv preprint arXiv:1606.07356.32369017

[B3] Agrawal A. Batra D. Parikh D. Kembhavi A. (2018). Don't just assume; look and answer: overcoming priors for visual question answering, in IEEE Conference on Computer Vision and Pattern Recognition, 4971–4980.

[B4] Anderson P. He X. Buehler C. Teney D. Johnson M. Gould S. (2018). Bottom-up and top-down attention for image captioning and visual question answering, in CVPR, Vol. 3.

[B5] Andreas J. Rohrbach M. Darrell T. Klein D. (2016a). Learning to compose neural networks for question answering. arXiv [preprint] arXiv:1601.01705.

[B6] Andreas J. Rohrbach M. Darrell T. Klein D. (2016b). Neural module networks, in CVPR.

[B7] Antol S. Agrawal A. Lu J. Mitchell M. Batra D. Lawrence Zitnick C. (2015). VQA: Visual question answering, in ICCV, 2425–2433.

[B8] Bahdanau D. Cho K. Bengio Y. (2014). Neural machine translation by jointly learning to align and translate. arXiv [preprint] arXiv:1409.0473.

[B9] Ben-Younes H. Cadene R. Cord M. Thome N. (2017). Mutan: Multimodal tucker fusion for visual question answering, in ICCV, Vol. 3.

[B10] Fukui A. Park D. H. Yang D. Rohrbach A. Darrell T. Rohrbach M. (2016). Multimodal compact bilinear pooling for visual question answering and visual grounding. arXiv [preprint] arXiv:1606.01847.

[B11] Goyal Y. Khot T. Summers-Stay D. Batra D. Parikh D. (2017). Making the V in VQA matter: Elevating the role of image understanding in visual question answering, in CVPR, Vol. 1.

[B12] He K. Zhang X. Ren S. Sun J. (2016). Deep residual learning for image recognition, in CVPR, 770–778.

[B13] Hu R. Andreas J. Darrell T. Saenko K. (2018). Explainable neural computation via stack neural module networks, in ECCV, 53–69.

[B14] Hu R. Andreas J. Rohrbach M. Darrell T. Saenko K. (2017). Learning to reason: end-to-end module networks for visual question answering, in Proceedings of the IEEE International Conference on Computer Vision, 804–813.

[B15] Hudson D. A. Manning C. D. (2019). GQA: A new dataset for real-world visual reasoning and compositional question answering. arxiv [Preprint] arXiv:1902.09506.

[B16] Jiang Y. Natarajan V. Chen X. Rohrbach M. Batra D. Parikh D. (2018). Pythia v0. 1: the winning entry to the VQA challenge 2018. arXiv [preprint] arXiv:1807.09956.

[B17] Johnson J. Hariharan B. van der Maaten L. Fei-Fei L. Zitnick C. L. Girshick R. (2017a). Clevr: a diagnostic dataset for compositional language and elementary visual reasoning, in CVPR, 1988–1997.

[B18] Johnson J. Hariharan B. van der Maaten L. Hoffman J. Fei-Fei L. Zitnick C. L. (2017b). Inferring and executing programs for visual reasoning, in ICCV, 3008–3017.

[B19] Kafle K. Kanan C. (2017). An analysis of visual question answering algorithms. arxiv [Preprint] arXiv:1703.09684.

[B20] Kazemi V. Elqursh A. (2017). Show, ask, attend, and answer: a strong baseline for visual question answering. arXiv [preprint] arXiv:1704.03162.

[B21] Kim J.-H. Jun J. Zhang B.-T. (2018). Bilinear attention networks. arXiv [preprint] arXiv:1805.07932.

[B22] Kinga D. Adam J. B. (2015). A method for stochastic optimization, in ICLR, Vol. 5.17802934

[B23] Lin T.-Y. Maire M. Belongie S. Hays J. Perona P. Ramanan D. (2014). Microsoft coco: common objects in context, in ECCV, 740–755.

[B24] Lu J. Yang J. Batra D. Parikh D. (2016). Hierarchical question-image co-attention for visual question answering, in NIPS, 289–297.

[B25] Mascharka D. Tran P. Soklaski R. Majumdar A. (2018). Transparency by design: closing the gap between performance and interpretability in visual reasoning, in CVPR, 4942–4950.

[B26] Noh H. Hongsuck Seo P. Han B. (2016). Image question answering using convolutional neural network with dynamic parameter prediction, in CVPR, 30–38.

[B27] Patro B. Namboodiri V. P. (2018). Differential attention for visual question answering, in CVPR, 7680–7688.

[B28] Pennington J. Socher R. Manning C. (2014). Glove: Global vectors for word representation, in EMMNLP, 1532–1543.

[B29] Ren S. He K. Girshick R. Sun J. (2015). Faster R-CNN: towards real-time object detection with region proposal networks, in NIPS, 91–99. 27295650 10.1109/TPAMI.2016.2577031

[B30] Russakovsky O. Deng J. Su H. Krause J. Satheesh S. Ma S. (2015). Imagenet large scale visual recognition challenge. Int. J. Comput. Vis. 115, 211–252. 10.1007/s11263-015-0816-y

[B31] Simonyan K. Zisserman A. (2014). Very deep convolutional networks for large-scale image recognition. arXiv [preprint] arXiv:1409.1556.

[B32] Tan H. Bansal M. (2019). Lxmert: Learning cross-modality encoder representations from transformers. arXiv preprint arXiv:1908.07490.

[B33] Vedantam R. Desai K. Lee S. Rohrbach M. Batra D. Parikh D. (2019). Probabilistic neural symbolic models for interpretable visual question answering. arxiv [Preprint] arXiv:1902.07864.

[B34] VQA (2016). VQA Challenge Leaderboard. Available online at: http://www.visualqa.org; http://evalai.cloudcv.org

[B35] Xu H. Saenko K. (2016). Ask, attend and answer: exploring question-guided spatial attention for visual question answering, in ECCV, 451–466.

[B36] Yang Z. He X. Gao J. Deng L. Smola A. (2016). Stacked attention networks for image question answering, in CVPR, 21–29.

[B37] Yu Z. Yu J. Fan J. Tao D. (2017). Multi-modal factorized bilinear pooling with co-attention learning for visual question answering, in ICCV, Vol. 3.29993847

